# Identification of QTNs and Their Candidate Genes for Boll Number and Boll Weight in Upland Cotton

**DOI:** 10.3390/genes15081032

**Published:** 2024-08-05

**Authors:** Xiaoshi Shi, Changhui Feng, Hongde Qin, Jingtian Wang, Qiong Zhao, Chunhai Jiao, Yuanming Zhang

**Affiliations:** 1 College of Plant Science and Technology, Huazhong Agricultural University, Wuhan 430070, China; shixiaoshi@webmail.hzau.edu.cn (X.S.); wangjingtian@webmail.hzau.edu.cn (J.W.); zq_08@webmail.hzau.edu.cn (Q.Z.); 2 Institute of Industrual Crops, Hubei Academy of Agricultural Sciences, Wuhan 430064, China; fch158@hbaas.ac.cn (C.F.); qinhongde@hbaas.ac.cn (H.Q.); 3 Hubei Academy of Agricultural Sciences, Wuhan 430064, China

**Keywords:** upland cotton, boll number, boll weight, GWAS, 3VmrMLM

## Abstract

Genome-wide association study (GWAS) has identified numerous significant loci for boll number (BN) and boll weight (BW), which play an essential role in cotton (*Gossypium* spp.) yield. The North Carolina design II (NC II) genetic mating population exhibits a greater number of genetic variations than other populations, which may facilitate the identification of additional genes. Accordingly, the 3VmrMLM method was employed for the analysis of upland cotton (*Gossypium hirsutum* L.) in an incomplete NC II genetic mating population across three environments. A total of 204 quantitative trait nucleotides (QTNs) were identified, of which 25 (24.75%) BN and 30 (29.13%) BW QTNs were of small effect (<1%) and 24 (23.76%) BN and 20 (19.42%) BW QTNs were rare (<10%). In the vicinity of these QTNs, two BN-related genes and two BW-related genes reported in previous studies were identified, in addition to five BN candidate genes and six BW candidate genes, which were obtained using differential expression analysis, gene function annotation, and haplotype analysis. Among these, six candidate genes were identified as homologs of Arabidopsis genes. The present study addresses the limitation of heritability missing and uncovers several new candidate genes. The findings of this study can provide a basis for further research and marker-assisted selection in upland cotton.

## 1. Introduction

Cotton is a major cash crop, and the yield of cotton is crucial in maintaining the stability of the global textile industry’s supply chain and fostering the growth of ancillary industries [1,2]. As living standards increase, the demand for cotton fiber across a range of sectors is rising in parallel. The cultivation of high-yielding cotton varieties remains a central objective in both cotton breeding programmes and agricultural practices [3].

The boll number (BN) and boll weight (BW) are significant components of cotton lint yield and have been demonstrated to be quantitative traits that are polygenically controlled [4]. A substantial number of genes influencing cotton yield-related traits have been identified. *GhLYI-A02* and *GhLYI-D08* have been demonstrated to influence lint percentage and boll number per plant [5], *GhCEN* has been shown to influence boll number [6], while *GhADF1* has been found to influence boll number and boll weight [7]. Based on the above findings, numerous studies have employed marker-assisted breeding to refine cotton breeding programmes [8,9]. Consequently, the identification and utilization of related genes provides an effective approach to enhancing cotton yield.

Genome-wide association study (GWAS) is an effective genetic method for the identification of associations between genetic markers and quantitative traits [10,11]. It can rapidly and precisely identify quantitative trait loci (QTLs) and provide detailed information that contributes to the identification and understanding of quantitative trait nucleotides (QTNs) and candidate genes, thereby enhancing the precision of genetic studies [12,13]. GWAS has been extensively employed to identify genes associated with a range of cotton traits, including *GhZF14*, which affects fiber length [14], and *GhRD2* and *GhNAC4*, which affect cold resistance [15], demonstrating the potential for improving agronomic traits. These findings have laid a crucial theoretical foundation for the development of superior cotton varieties, especially those with the potential for increased yield [16,17,18,19,20].

At present, numerous GWAS methods and corresponding software packages have been developed [21,22,23,24,25,26,27,28]. Li et al. [28] established a 3VmrMLM model for detecting and estimating all the possible effects of QTNs while controlling for all the possible polygenic backgrounds, and they developed the IIIVmrMLM software package to dissect the genetic basis of complex traits [29]. This method reduces the number of variance components in the mixed linear model and increases the power of identifying the genes with allelic substitution effect close to zero, small effects, and rare allele frequency [28,29,30], thereby enabling comprehensive analysis of the genetic basis of quantitative traits.

The North Carolina II (NC II) genetic mating design comprises two parental groups: one comprising the father and the other comprising the mother. The two groups cross, resulting in the production of hybrid offspring [31]. The NC II population’s genetic diversity is enhanced through the identification of genetic markers associated with target traits, which may facilitate the identification of additional genes [32,33,34]. However, most of the research conducted on the NC II population employed conventional GWAS methods that are not optimal for the NC II population, which may have resulted in the loss of heritability. Thus, the 3VmrMLM method was used to analyze the data in the NC II design.

In this study, we employed 3VmrMLM to associate BN and BW phenotypes in three environments with 3,480,274 single nucleotide polymorphisms (SNPs) in an incomplete NC II population of upland cotton. The objective of this study was to identify candidate genes for BN and BW to provide a foundation for further research and marker-assisted selection for cotton yield.

## 2. Results

### 2.1. Phenotypic Variation and Analysis of Variance

**Figure 1:**
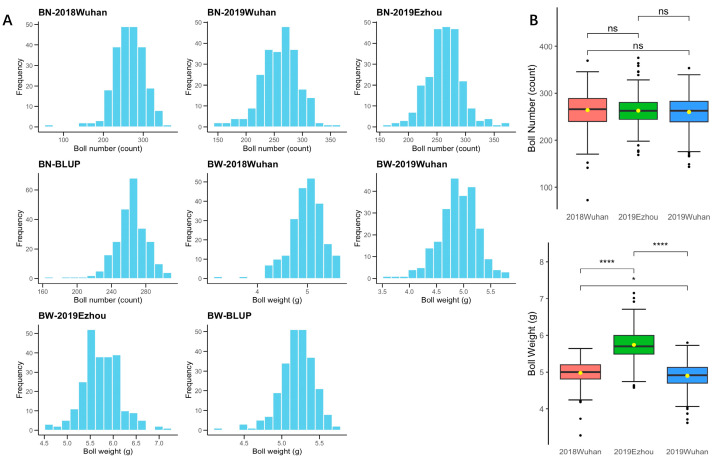
Analysis of boll number (BN) and boll weight (BW) phenotypes in 240 upland cotton cultivars/lines. (**A**) Histograms of phenotypic distribution; (**B**) Box plots. *, and ****: the 0.05, and 0.0001 probability levels of significance, respectively; ns: no significant difference at the 0.05 probability level. Yellow dots: average value.

A statistical analysis, comprising descriptive statistics and analysis of variance, was performed on the phenotypic data of BN and BW of upland cotton using R v4.3.3 software (Table 1; Figure 1A,B). The mean BN ranged from 259.55 to 264.19 (counts), while the mean BW ranged from 4.88 g to 5.74 g, respectively. The standard deviation for BN was between 31.74 and 38.28, while that for BW was between 0.34 and 0.41, respectively. The BN and BW phenotypic distributions were continuous and slightly skewed (Figure 1A), being quantitative traits. The coefficients of variation ranged from 12.05% to 14.49% for BN and from 6.85% to 7.29% for BW. The coefficients of variation of best linear unbiased prediction (BLUP) values for both traits were smaller due to the removal of environmental variation. The broad-sense heritabilities for BN and BW were 69.79% and 74.55%, respectively, with mean BLUP values of 262.40 counts and 5.20 g, respectively. These results indicated that the two traits, BN and BW, exhibited high inter-individual phenotypic variability, rendering this population suitable for GWAS.

BW exhibited highly significant differences among the three environments (*F* = 59.83~377.80; *p* < 0.001), and BW in 2019 Ezhou (5.74 ± 0.41 g) was significantly higher than 2018 Wuhan (4.98 ± 0.34 g) and 2019 Wuhan (4.88 ± 0.36 g). However, the differences in three environments for BN were not significant (F = 0.83; *p* = 0.437), as illustrated in Figure 1B.

### 2.2. Identification of QTNs for Boll Number and Boll Weight Using the Single Environment Analysis Module of 3VmrMLM

**Figure 2:**
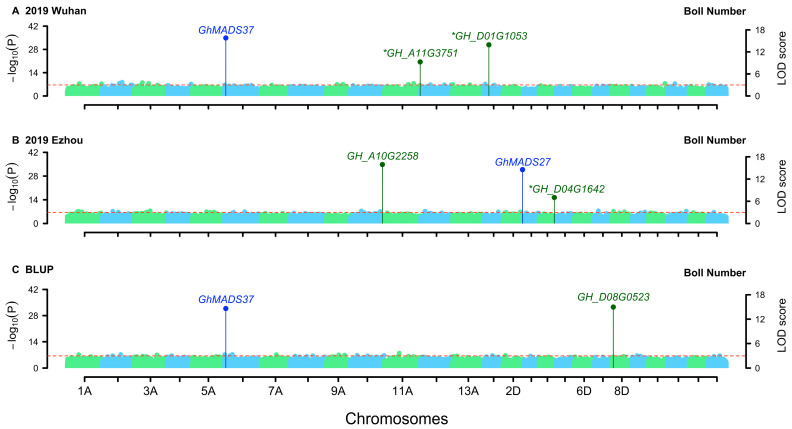
Manhattan plots for boll number in upland cotton. Trait-related genes around QTNs were marked with blue, while candidate genes around QTNs were marked with dark green. The key candidate genes were marked by asterisk (*). (**A**) 2019 Wuhan; (**B**) 2019 Ezhou; (**C**) BLUP.

**Figure 3:**
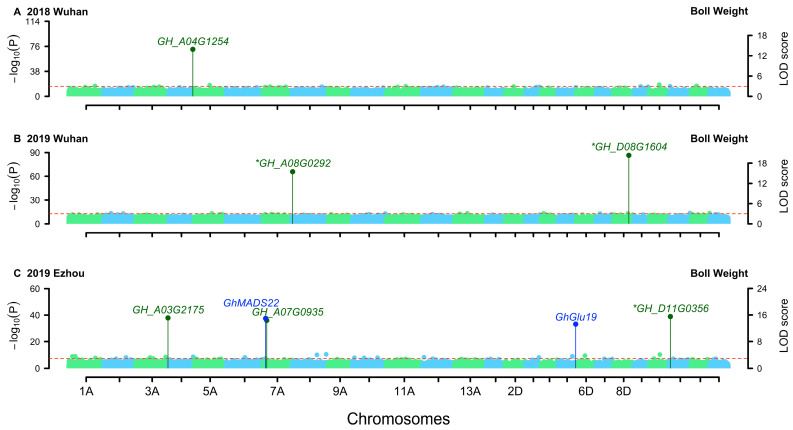
Manhattan plots for boll weight in upland cotton. Trait-related genes around QTNs were marked with blue, while candidate genes around QTNs were marked with dark green. The key candidate genes were marked by asterisk (*). (**A**) 2018 Wuhan; (**B**) 2019 Wuhan; (**C**) 2019 Ezhou.

GWAS was conducted using single environment analysis module of 3VmrMLM on the genotypic data of 240 upland cotton cultivars/lines, along with their BN and BW phenotypic data in the three environments and their BLUP values. A total of 101 QTNs were identified for BN (Figure 2) and 103 for BW (Figure 3; Table 2). The additive effects of QTNs for BN ranged from −34.75 to 33.14, and the dominant effects ranged from −30.09 to 26.11, explaining 0.18% to 7.87% of the total phenotypic variance. The additive effects of QTNs for BW ranged from −0.60 to 0.35, and the dominant effects ranged from −0.46 to 0.54, explaining 0.21% to 8.48% of the total phenotypic variance.

The total variance explained by the QTLs in three environments were 638.13, 503.94, and 137.73 for BN, and 0.038, 0.054, and 0.097 for BW, respectively, which were 69.79% (BN) and 74.55% (BW) to the sum of QTN size (Table 3). These results indicate that a significant proportion of trait heritabilities (48.12% for BN and 59.64% for BW on average) is explained by the GWAS results.

Three QTNs were identified repeatedly in multiple datasets. *Marker639503* was identified in both 2019 Wuhan and BLUP values for BN, while *marker1655415* and *marker2032522* were detected in both 2019 Ezhou and BLUP values for BW.

### 2.3. Candidate Gene Prediction for Boll Number and Boll Weight

Candidate genes were predicted by differential expression analysis, Gene Ontology (GO) annotation, and haplotype analysis. The cotton ovary contains numerous ovules, with the elongated epidermal cells of each ovule differentiating to form cotton fibers. The development of the ovule affects the development of the cotton boll, which in turn has an important effect on BN and BW [35]. For the remaining QTNs without the related genes reported in previous studies, their nearby differentially expressed genes (DEGs) in ovules were analyzed. A total of 79, 110, 50, and 84 DEGs were identified to be around significant and suggested QTNs for BN in the 2018 Wuhan, 2019 Wuhan, 2019 Ezhou, and BLUP datasets, respectively, while 59, 36, 148, and 142 DEGs were identified as being approximately significant and suggested QTNs for BW in the 2018 Wuhan, 2019 Wuhan, 2019 Ezhou, and BLUP datasets, respectively. Subsequent GO annotation of these DEGs yielded 37 and 48 potential candidate genes for BN and BW, respectively.

The 2 kb sequence upstream of the transcription start site is recognised as the promoter region, and SNPs within this region and potential candidate genes have been extracted [36]. Haplotype analysis revealed the significant associations between five candidate genes and BN (Figure 2) and between six candidate genes and BW (Figure 3; Table 3).

**Table 3:** Candidate genes in proximity to QTNs for boll number (BN) and boll weight (BW) in upland cotton. I: 2018 Wuhan; II: 2019 Wuhan; III: 2019 Ezhou.

Trait	Chr	Posi (bp)	LOD Scores				r^2^ (%)	Gene Differential Expression Analysis			*p*-Value in Haplotype Analysis	GO Annotation Analysis			
			I	II	III	BLUP		Gene_ID	log_2_ (Fold Change)	*p*-Value		GO_ID	GO_Name	E-Value	Reference
BN	A10	108368842			15.92		4.22	*GH_A10G2258*	3.77	7.75 × 10^−13^	5.53 × 10^−3^	GO:0009739	response to gibberellin	<1.00 × 10^−300^	[37]
	A11	121669424		9.27			1.18	*GH_A11G3751*	4.48	3.07 × 10^−4^	1.27 × 10^−4^	GO:0009738	abscisic acid-activated signaling pathway	<1.00 × 10^−300^	[38]
	D01	15378670		13.94			1.52	*GH_D01G1053*	−2.08	1.73 × 10^−12^	1.38 × 10^−2^	GO:0009409	response to cold	<1.00 × 10^−300^	[39]
	D04	51658044			7.01		1.75	*GH_D04G1642*	−2.83	1.26 × 10^−10^	4.73 × 10^−2^	GO:0048481	plant ovule development	6.22 × 10^−150^	
	D08	5543570				14.97	1.28	*GH_D08G0523*	4.19	0	2.50 × 10^−3^	GO:0071215	cellular response to abscisic acid stimulus	<1.00 × 10^−300^	[38]
BW	A03	110026226			18.97		3.20	*GH_A03G2175*	−2.40	2.78 × 10^−3^	1.10 × 10^−2^	GO:0009737	response to abscisic acid	<1.00 × 10^−300^	[38]
	A04	81070268	13.91				2.37	*GH_A04G1254*	−3.70	3.33 × 10^−10^	1.44 × 10^−3^	GO:0009409	response to cold	3.98 × 10^−169^	[39]
	A07	12833357			14.36		1.98	*GH_A07G0935*	−2.47	8.52 × 10^−7^	5.03 × 10^−4^	GO:0009651	response to salt stress	5.38 × 10^−219^	[40]
	A08	2669815		27.56			8.48	*GH_A08G0292*	2.12	1.55 × 10^−2^	2.23 × 10^−4^	GO:0009738	abscisic acid-activated signaling pathway	1.31 × 10^−143^	[38]
	D08	51949701		149.89			1.81	*GH_D08G1604*	2.56	5.12 × 10^−3^	2.79 × 10^−2^	GO:0009409	response to cold	4.27 × 10^−273^	[39]
	D11	2810467			15.53		2.01	*GH_D11G0356*	−3.67	2.15 × 10^−6^	2.72 × 10^−3^	GO:0009409	response to cold	8.41 × 10^−278^	[39]

### 2.4. Alignment of Candidate Genes and Arabidopsis Homologous Sequences

The blast tool of TAIR (https://www.arabidopsis.org, accessed on 19 May 2024) was employed to analyze the homologous genes of candidate genes in *Arabidopsis thaliana*. A comparison of Arabidopsis homologous genes revealed three and three key candidate genes for BN and BW, respectively (Table 4).

## 3. Discussion

### 3.1. QTNs Detection in This Study Recovered Some Heritability

In this study, 3VmrMLM was applied to analyze the BN and BW of cotton incomplete NC II data. The total genetic variances explained by all the QTLs for BN and BW were 33.58% and 44.46%, respectively, on average in the three environments, accounting for 48.12% BN and 59.64% BW heritabilities, respectively (Table 1 and Table 2). As we know, quantitative traits are controlled by genes and gene-by-gene interactions and modified by environments and gene-by-environment interactions, where these genes include some major genes and many polygenes each with a relatively small effect. Based on our study in Wang et al. [30], it is difficult for conventional GWAS methods to identify small allele substitution effects, dominant effects, and rare loci. In this study, only the QTNs with additive and dominant effects were identified. Although the loss of heritability of complex traits is common in GWAS [47], the above proportions (48.12% and 59.64%) are relatively high, indicating that this study recovered some heritability. More importantly, a total of four trait-related genes and six candidate genes around 204 QTNs were identified by the new method (Figure 2 and Figure 3), offering novel insights for cotton breeding and GWAS in incomplete NC II populations.

### 3.2. Related Genes around QTNs for Boll Number and Boll Weight

Previous studies have reported some genes associated with BW and BN. Trait-related genes were identified within a 500 kb region upstream and downstream of the QTNs [36]. Flower bud differentiation represents the developmental basis of cotton bud appearance, flowering, and boll setting and is an important factor affecting cotton yield [48]. Two BN-related and two BW-related genes reported in previous studies were identified for BN and BW, respectively (Table 5; Figure 2 and Figure 3). *GhMADS37* is highly expressed in apical buds and flowers. *GhMADS27* is highly expressed in flowers [49]. Overexpression of *GhMADS22* delays senescence and abscission of floral organs and is responsive to abscisic acid [50]. In addition, the inhibition of *GhGlu19* expression significantly increases BW, BN, as well as lint percentage, and also enhances seed vigor, resulting in a significant increase in cotton yield [51].

### 3.3. The Interaction of Boll Number and Boll Weight with Environment in Upland Cotton

The phenotypic data for BN exhibited no significant divergence across the three environments, whereas those for BW did (Figure 1B), suggesting that BW may be more susceptible to environmental influences than BN. A multi-environmental joint analysis of the phenotypic data for BW in the three environments was therefore conducted using 3VmrMLM (Table S2). However, only one potential candidate gene-environment interaction was identified, namely *GH_D09G2342*. The growth of cotton bolls is significantly influenced by ambient temperature [39], and *GH_D09G2342* plays a role in regulating the response of upland cotton to heat, which may be involved in interactions between upland cotton and the environment.

In this study, several candidate genes for BW have also been identified as being associated with environmental responses. *GH_A03G2175* regulates abscisic acid response, *GH_A07G0935* regulates response to salt stress, and *GH_A04G1254*, *GH_D08G1604*, and *GH_D11G0356* regulate response to cold. The aforementioned results indicate that these genes are likely to play a role in the interaction between BW and the environment.

### 3.4. New Candidate Genes for Boll Number and Boll Weight in Upland Cotton

Some candidate genes are considered to be key, such as *GH_D04G1642*, which is involved in ovule development. Its homologue in *Arabidopsis thaliana*, *TT16*, regulates ovule development, and *tt16* mutants exhibit reduced endosperm development following fertilisation [43]. The potential application of such gene in marker-assisted selection could facilitate the development of cotton varieties with enhanced yield stability under diverse environmental conditions.

Furthermore, some candidate genes may regulate BN and BW through phytohormones. *GH_A11G3751* is involved in the abscisic acid signalling pathway, and its Arabidopsis homologue, *CPK11*, positively regulates calcium-mediated abscisic acid signalling. Seedlings overexpressing *CPK11* exhibit a significant increase in sensitivity to abscisic acid [41], a trait that could be harnessed to develop cotton varieties with enhanced resilience to stress. *GH_A08G0292* is involved in the abscisic acid signalling pathway, and its homologue, *LEC1*, is crucial for development of *Arabidopsis* embryo. Together with *ABI3*, *LEC2*, and *FUS3*, it constitutes the *LAFL* regulatory network, which controls key processes in seed development and maturation, with *ABI3* playing a supporting role in the abscisic acid response [44]. Regulation of these genes could lead to enhanced cotton yield and quality.

In addition to the aforementioned genes, other candidate genes are involved in the response to cold stimuli, indicating that multiple genes may influence cotton boll development through the response to temperature. *GH_D01G1053* may be a valuable marker for the selection of cotton with enhanced cold tolerance, given that its homologue, *CAT3*, is involved in the regulation of the biological clock in *Arabidopsis* and is sensitive to changes in temperature and light conditions, with expression peaking especially in the early evening hours. In contrast, flowering time is significantly prolonged in the *cat3* mutant [42]. The homologue of *GH_D08G1604*, *LCBK2*, is involved in PHS-P formation in *Arabidopsis*. The *lcbk2* mutant exhibits a blocked PHS-P formation under cold stimulation, indicating an important role for *LCBK2* in cold signalling. *GH_D08G1604* could be a key gene for enhancing cold tolerance in upland cotton through marker-assisted selection [45]. Furthermore, *GH_D11G0356*, which has an Arabidopsis homologue, *MDH*, that displays increased expression under cold conditions, may also serve as a potential target for enhancing cold resistance in upland cotton [46].

The above genes may account for the sensitivity of upland cotton boll development to environmental changes. Future studies should aim to validate the role of these candidate genes in the environmental response of BW and explore their specific mechanisms of action under various environmental conditions. Furthermore, studies should be expanded to include yield-related traits, as well as other traits, and their interactions with environmental factors.

### 3.5. Comparison of GWAS Results with and without PCA

Incorporating the principal component analysis (PCA) result as the population structure identified 19, 24, 19, and 20 QTNs in BN and 19, 20, 20, and 20 QTNs in BW (Table S4). Only in 2019 Ezhou, 2 BN- and 2 BW-related genes were identified (Table S5), indicating that the inclusion of PCA as a population structure has a negative impact on the accuracy of GWAS results. Therefore, PCA was not considered in this study.

## 4. Materials and Methods

### 4.1. Plant Material and Phenotypic Data

**Figure 4:**
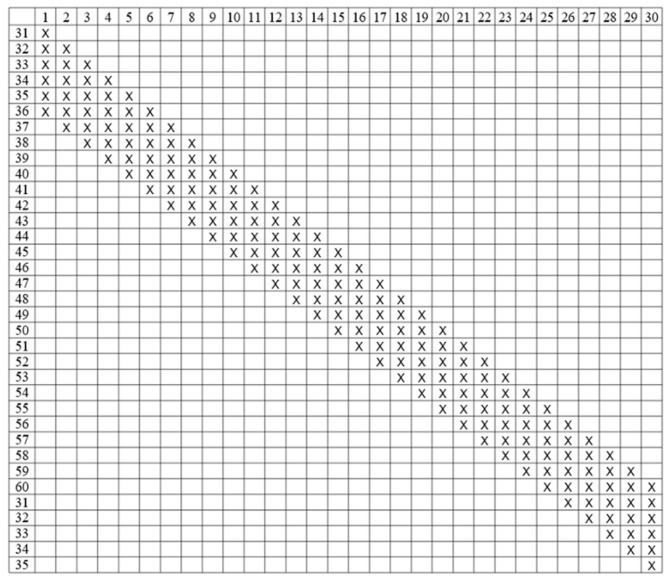
Parental combinations of 60 upland cotton cultivars in partial NC II genetic mating design. X: the cross was made.

In this study, a collection of 60 upland cotton cultivars, sourced from diverse geographical regions, were selected to serve as parental lines (Table S6). Specifically, the first 30 cultivars were designated as male parents, while cultivars numbered 31 to 60 were utilized as female parents. In the Figure 4, each X corresponds to an F_1_ hybrids progeny, and its corresponding row and column are the numbers of its paternal and maternal, respectively. These hybrid combinations were meticulously designed in Hainan during 2017. The above 60 parents, along with their 180 F_1_ hybrid progeny, which collectively represent 240 upland cotton cultivars/lines, were subsequently planted.

To evaluate the performance of upland cotton under varied conditions, a total of 240 upland cotton cultivars/lines were cultivated in three distinct experimental settings. The seedling raising of these cultivars/lines commenced on 17 April 2018 and 2019 at the Wuhan Experimental Base of the Institute of Economic Crops, Hubei Academy of Agricultural Sciences, followed by transplanting on 11 May. Furthermore, in 2019, a parallel planting was conducted at the Ezhou Experimental Base of the same institute, utilising direct seeding on 25 April. In all environments, a consistent randomised block design was implemented, featuring single-row plots, each with an area of 3.34 m^2^ and row spacing of 0.76 m, and triplicate repetitions for each cultivar/line.

In both 2018 and 2019, a comprehensive survey was conducted during the period around 20 September to enumerate the total boll count in each plot. From the central portion of each row, a sample of 50 bolls that had fluffed normally was collected for further analysis. Subsequently, the average weight of this boll sample was calculated.

### 4.2. Statistical Analysis and Analysis of Variance of Phenotypic Data

Descriptive statistical analysis and variance analysis were conducted to evaluate the performance of upland cotton phenotypic data across different environments. The statistical measures, including maximum, minimum, mean, standard deviation (SD), coefficient of variation (CV), kurtosis, and skewness, for BN and BW in the 2018 Wuhan, 2019 Wuhan, and 2019 Ezhou datasets were calculated using the R package psych v2.4.3.

The R package lme4 v1.1-35.2 was employed to calculate the BLUP values of each individual and the heritability of each trait. The BLUP values were calculated using the model: $y_{ijk}=\mu +G_{k}+E_{i}+R_{j\left(i\right)}+GE_{ik}+\epsilon_{ijk}$, where $y_{ijk}$ is the observed value of the *j*th repetition of the *k*th cultivar/line in the *i*th environment, *µ* is the overall mean, $G_{k}$ is the fixed effect of the *k*th cultivar/line, $E_{i}$ is the random effect of the *i*th environment, $R_{j\left(i\right)}$ is the random effect of the *j*th repetition nested in the *i*th environment, $GE_{ik}$ is the random effect of the interaction between the *i*th environment and *k*th cultivar/line, and $\epsilon_{ijk}$ is the residual error. The heritability was calculated using the following formula: $h_{B}^{2}=\frac{\sigma_{g}^{2}}{\sigma_{g}^{2}+\sigma_{e}^{2}/l}\times 100\%$, where $\sigma_{g}^{2}$ represents the genetic variance, $\sigma_{e}^{2}$ represents the error variance, *l* represents the number of environments.

The R function *aov* was employed to identify significant differences in BN and BW over the different environments. Subsequently, the R package ggplot2 v3.5.0 was employed to generate box plots visualizing these differences.

### 4.3. Genotype Data

Genomic DNA was extracted from young fresh leaves of 60 upland cotton parents using the CTAB method, as previously described [52]. The raw sequencing data were base-called to generate raw reads, which were then subjected to quality control using the fastp v0.23.4 software [53] to produce clean reads suitable for further analysis. The high-quality reads obtained after filtering were aligned to the reference genome using the BWA v0.7.17 software [54], and the poor-quality reads were discarded. The resulting SAM files were sorted, and PCR duplicates were identified and removed using the Picard tool. The sorted BAM files were then indexed to facilitate subsequent analysis. Read counts for each sample were determined using the SAMtools v1.11 software [55]. Population-wide SNP and Indel detection and genotyping were performed using the GATK v4.1.3.0 software [56].

Subsequently, a paired-end (PE) library was constructed for each sample according to the Illumina Library Construction Protocol. This involved randomly shearing the genomic DNA into fragments of 300–500 bp, repairing the ends of these fragments, and ligating adapters to create the sequencing junctions. Subsequently, DNA clusters were prepared on sequencing chips, and the libraries were sequenced online using the Illumina HiSeq 4000 platform with a PE150 sequencing strategy, yielding comprehensive genomic data for each cultivar.

This process yielded 3,480,274 SNP markers for each upland cotton cultivar. The genotypes of the 180 F_1_ hybrids were inferred based on the genotypes of the parental cultivars. The sequencing of the upland cotton cultivars was completed by Wuhan Gooalgene Technology Co. (Wuhan, China).

### 4.4. Multi-Locus GWAS

The R package IIIVmrMLM [29] was employed to conduct GWAS on 3,480,274 SNP markers and phenotypic values of both BN and BW, along with their BLUP values across 3 environments, in 240 upland cotton cultivars/lines. All parameters were set to their default values. The K-matrix was calculated using the IIIVmrMLM v1.0 software; the population structure was not included in the mixed model. Significant and suggested QTNs were determined by *p* ≤ 0.05/m and LOD ≥ 3.0, respectively, where *m* is the number of markers [28].

### 4.5. Identification of Related Genes for Boll Number and Boll Weight

The genes related to the BN and BW have been summarised through gene annotations available on CottonFGD (https://cottonfgd.org/, accessed on 22 April 2024) and CottonGVD (https://db.cngb.org/cottonGVD, accessed on 22 April 2024), as well as by reviewing relevant literature. Genes around QTNs were mined within a 500 kb region upstream and downstream [36], using the reference genome of upland cotton from the GCGI (http://cotton.hzau.edu.cn, accessed on 22 April 2024).

### 4.6. Candidate Genes Prediction for Boll Number and Boll Weight

The ovule transcriptome data between upland cotton TM-1 and sea island cotton Hai7124 (accession number: GSE119184) [57] were used to perform differential expression analysis using R package DEGseq v1.48 [58]. The threshold for significance was set at *p* < 0.05 and $\left|\log_{2}\left(Fold\;Change\right)\right|$ > 2 [59]. The DEGs from ovules at 10 DPA and 20 DPA ovules were pooled for subsequent analyses. The upland cotton genomic data in CottonFGD (https://cottonfgd.net/about/download.html, accessed on 14 April 2024) was used as a reference genome.

The protein sequence information of the upland cotton TM-1 genome was downloaded from IBI (http://ibi.zju.edu.cn/, accessed on 14 May 2024). The protein sequences of the differentially expressed genes in the ovules from the previous step were extracted using TBtools [60], and were then annotated using AgBase (https://agbase.arizona.edu, accessed on 14 May 2024) [61] for GO annotation, with significant e-values set to $10^{-50}$, to identify genes whose biological processes are associated with bud differentiation or flowering, and thus potentially eligible as potential candidate genes.

Sequences 2 kb upstream of the transcription start site were considered as promoter regions and SNPs were extracted within potential candidate genes and their promoter regions [36]. Significant SNP information within the above potential candidate genes as well as 2 kb upstream was extracted using R v4.3.3. ANOVA was performed using the R function *aov*, and the significant level was set at 0.05.

### 4.7. Homologous Genes in Arabidopsis thaliana

The candidate gene sequences of upland cotton were extracted from CottonFGD (https://cottonfgd.net/about/download.html, accessed on 14 May 2024) and then entered into TAIR (https://www.arabidopsis.org) for comparison with their homologous genes in *Arabidopsis thaliana*.

## 5. Conclusions

3VmrMLM was employed to associate the BN and BW phenotypes in three environments and their BLUP values with 3,480,274 SNPs in 240 upland cotton cultivars/lines. A total of 204 QTNs explaining 0.18% to 8.48% of the phenotypic variance were identified to be associated with BN and BW in upland cotton. Of the identified QTNs, 25 (24.75%) BN and 30 (29.13%) BW QTNs were small (<1%), while 24 (23.76%) BN and 20 (19.42%) BW QTNs were rare (<10%). A total of four trait-related genes, *GhMADS37*, *GhMADS27*, *GhMADS22*, and *GhGlu19*, were identified in the vicinity of the QTNs. Six key candidate genes were identified: *GH_A11G3751*, *GH_D01G1053*, *GH_D04G1642*, *GH_A08G0292*, *GH_D08G1604*, and *GH_D11G0356*. This study addresses the challenge of missing heritability and expands the genetic repertoire available for marker-assisted selection. The newly identified candidate genes provide a solid foundation for advancing research and for the improvement of cotton yield through marker-assisted breeding strategies.

## Figures and Tables

**Table 1:** The descriptive statistics of phenotypic data for boll number (BN) and boll weight (BW) in 240 upland cotton cultivars/lines.

Trait	Location	Max	Min	Mean	SD	Median	Kurtosis	Skewness	CV (%)	$h_{B}^{2}$ (%)
BN	2018 Wuhan	369.17	72.40	264.19	38.28	266.00	2.49	−0.63	14.49	69.79
	2019 Wuhan	353.50	143.33	259.55	34.29	262.20	0.71	−0.46	13.21	
	2019 Ezhou	375.67	168.67	263.46	31.74	263.17	1.22	0.35	12.05	
	BLUP	307.70	169.98	262.40	19.29	263.27	2.63	−0.83	7.35	
BW	2018 Wuhan	5.64	3.27	4.98	0.34	5.00	2.66	−0.89	6.85	74.55
	2019 Wuhan	5.80	3.62	4.88	0.36	4.90	0.87	−0.46	7.29	
	2019 Ezhou	7.15	4.57	5.74	0.41	5.70	0.91	0.25	7.18	
	BLUP	5.77	4.20	5.20	0.23	5.21	1.73	−0.70	4.35	

**Table 2:** Numbers of all the QTNs and their genetic variances and heritabilities for boll number (BN) and boll weight (BW) of upland cotton in different environments.

Trait	Environment	Numbers of QTNs	Total Genetic Variance Explained by the QTNs	Heritabilities of All the QTNs (%)
BN	2018 Wuhan	23	638.13	43.74
	2019 Wuhan	28	503.94	43.37
	2019 Ezhou	18	137.73	13.64
	BLUP	32		
BW	2018 Wuhan	21	0.038	32.87
	2019 Wuhan	20	0.054	42.05
	2019 Ezhou	35	0.097	58.46
	BLUP	27		

**Table 4:** Key candidate genes of boll number (BN) and boll weight (BW) in upland cotton and their homologous genes in *Arabidopsis thaliana*.

Trait	Candidate Genes in Cotton	Homologous Genes in *Arabidopsis thaliana*			
		Homology	Gene Name	E-Value	Reference
BN	*GH_A11G3751*	*AT1G35670*	*CPK11*	<1 × 10^−100^	[41]
	*GH_D01G1053*	*AT1G20620*	*CAT3*	<1 × 10^−100^	[42]
	*GH_D04G1642*	*AT5G23260*	*TT16*	3 × 10^−90^	[43]
BW	*GH_A08G0292*	*AT1G21970*	*LEC1*	7 × 10^−58^	[44]
	*GH_D08G1604*	*AT2G46090*	*LCBK2*	<1 × 10^−100^	[45]
	*GH_D11G0356*	*AT3G47520*	*MDH*	<1 × 10^−100^	[46]

**Table 5:** Related genes, reported in previous studies, around QTNs for boll number (BN) and boll weight (BW) in upland cotton. I: 2018 Wuhan; II: 2019 Wuhan; III: 2019 Ezhou. Trait-related genes with blue were repeatedly identified.

Trait	Chr	Position (bp)	LOD Scores				r^2^ (%)	Comparative Genomics Analysis		Reference
			I	II	III	BLUP		Related Genes	Distance (kb)	
BN	A06	4,326,968		36.29		6.28	0.66~2.72	*GhMADS37*	324.57	[49]
	D02	66,249,932			4.58		1.60	*GhMADS27*	27.16	[49]
BW	A07	9,022,998~9,339,027			8.26; 15.49		1.18~1.86	*GhMADS22*	76.59~392.61	[50]
	D05	61,069,275			13.26		1.66	*GhGlu19*	173.58	[51]

## Data Availability

The data used in this study and the original analysis results are available from the Online Tables or from the corresponding authors.

## Supplementary Materials

The following supporting information can be downloaded at: https://www.mdpi.com/article/10.3390/genes15081032/s1. Tables S1 and S2: Detailed information of QTNs and QEIs for BN and BW in upland cotton GWAS; Table S3: PCA of 240 upland cotton cultivars/lines; Tables S4 and S5: QTNs and trait related genes for BN and BW in upland cotton GWAS with PCA; Table S6. Names and origins of 60 parents.
